# Overweight worsens apoptosis, neuroinflammation and blood-brain barrier damage after hypoxic ischemia in neonatal brain through JNK hyperactivation

**DOI:** 10.1186/1742-2094-8-40

**Published:** 2011-04-25

**Authors:** Yi-Fang Tu, Yau-Sheng Tsai, Lan-Wan Wang, Hsin-Chieh Wu, Chao-Ching Huang, Chien-Jung Ho

**Affiliations:** 1 Institute of Clinical Medicine, National Cheng Kung University College of Medicine, Tainan, Taiwan; 2 Department of Emergency Medicine, National Cheng Kung University College of Medicine and Hospital, Tainan, Taiwan; 3 Department of Pediatrics, Chi Mei Medical Center, Tainan, Taiwan; 4 Department of Pediatrics, National Cheng Kung University College of Medicine and Hospital, Tainan, Taiwan

## Abstract

**Background:**

Apoptosis, neuroinflammation and blood-brain barrier (BBB) damage affect the susceptibility of the developing brain to hypoxic-ischemic (HI) insults. c-Jun N-terminal kinase (JNK) is an important mediator of insulin resistance in obesity. We hypothesized that neonatal overweight aggravates HI brain damage through JNK hyperactivation-mediated upregulation of neuronal apoptosis, neuroinflammation and BBB leakage in rat pups.

**Methods:**

Overweight (OF) pups were established by reducing the litter size to 6, and control (NF) pups by keeping the litter size at 12 from postnatal (P) day 1 before HI on P7. Immunohistochemistry and immunoblotting were used to determine the TUNEL-(+) cells and BBB damage, cleaved caspase-3 and poly (ADP-ribose) polymerase (PARP), and phospho-JNK and phospho-Bim_EL _levels. Immunofluorescence was performed to determine the cellular distribution of phospho-JNK.

**Results:**

Compared with NF pups, OF pups had a significantly heavier body-weight and greater fat deposition on P7. Compared with the NF-HI group, the OF-HI group showed significant increases of TUNEL-(+) cells, cleaved levels of caspase-3 and PARP, and ED1-(+) activated microglia and BBB damage in the cortex 24 hours post-HI. Immunofluorescence of the OF-HI pups showed that activated-caspase 3 expression was found mainly in NeuN-(+) neurons and RECA1-(+) vascular endothelial cells 24 hours post-HI. The OF-HI group also had prolonged escape latency in the Morris water maze test and greater brain-volume loss compared with the NF-HI group when assessed at adulthood. Phospho-JNK and phospho-Bim_EL _levels were higher in OF-HI pups than in NF-HI pups immediately post-HI. JNK activation in OF-HI pups was mainly expressed in neurons, microglia and vascular endothelial cells. Inhibiting JNK activity by AS601245 caused more attenuation of cleaved caspase-3 and PARP, a greater reduction of microglial activation and BBB damage post-HI, and significantly reduced brain damage in OF-HI than in NF-HI pups.

**Conclusions:**

Neonatal overweight increased HI-induced neuronal apoptosis, microglial activation and BBB damage, and aggravated HI brain damage in rat pups through JNK hyperactivation.

## Background

Hypoxic ischemia (HI) is a major cause of mortality and neurological disabilities in infants. Approximately 30-40% of infants with HI die at birth, and 20-40% of the survivors develop significant neurological deficits, including permanent neuromotor and cognitive impairment [1-3]. Obesity, which is associated with the metabolic syndrome, is an independent risk factor for stroke in adults [4,5]. Growing evidence indicates that obese adults suffer a higher risk of stroke, and may have a worse prognosis post-stroke than non-obese adults [4-6]. Similar to the obesity effect in adults, large-for-gestational age newborns who have above-average body weights at birth have higher incidences of birth complications, such as hyperinsulinemia and hypoglycemia, than appropriate-for-gestational age newborns [7]. However, it remains to be determined whether being overweight aggravates HI injury in neonatal brains.

Apoptosis is an important component of HI injury in neonatal brains. Activation of apoptotic pathways leads to activation of caspase-3 and poly (ADP-ribose) polymerase (PARP), which are maximally expressed in the neonatal period [2,3]. Substantial evidence has documented that activated microglia are the hallmark of neuroinflammation and exacerbate brain injury through production of pro-inflammatory cytokines [3,8]. The blood-brain barrier (BBB) restricts the access of molecules and cells into the brain, and its disruption in neonatal brains has been linked to the severity of HI injury [2,9]. Therefore, neuronal apoptosis, neuroinflammation, and BBB damage may account for the higher susceptibility of the developing brain to HI injury [2,3,8,9]. It remains unclear whether being overweight aggravates HI injury by magnifying neuronal apoptosis, microglial activation and BBB damage in the neonatal brain.

c-Jun N-terminal kinase (JNK), a family of serine/threonine protein kinases of the mitogen-activated protein kinase group, has recently emerged as an important regulator of insulin resistance in obesity [10]. JNKs are important stress responsive kinases that are activated by various forms of insults, including oxidative stress and ischemia. JNK activation precedes cell death by apoptosis and inflammation in many cell types [11]. Whether being overweight aggravates apoptosis, microglia activation and BBB leakage after HI, and thereby worsening brain damage through JNK hyperactivation in neonatal brains remains unknown.

Reducing litter size and increasing milk availability during the suckling period has been utilized to induce overweight juvenile rats [12,13]. Rat pups from small litters develop excess body weight and adipose tissue in the early postnatal period. Using this rat model of reducing the litter size to induce overweight pups, we tested the hypothesis that JNK hyperactivation as a result of neonatal overweight aggravates HI-induced neuronal apoptosis, microglial activation and BBB injury, and exaggerates HI brain damage in neonatal rats.

## Materials and methods

### Animals

This study was approved by our university's Animal Care Committee. Sprague-Dawley rat pups were housed with a 12/12-h light/dark schedule in a temperature and humidity controlled room. The overweight (OF) rat pups were induced by culling the litter size to 6 pups per dam from postnatal (P) day 1 until weaning, and the control (NF) pups by keeping the litter size at 12. Only male pups were used for this study.

### Hypoxic-ischemia brain injury in rat pups

On P7, rat pups were anesthetized with 2.5% halothane, followed by permanent ligation of the right common carotid artery with 5-0 surgical silk. After surgery, the pups were returned to their dams for a 1-hour recovery period before 2 hours of hypoxia. During hypoxia, the pups were placed in air-tight 500-ml containers with 37°C humidified 8% oxygen (balance, nitrogen) [14,15]. Rectal temperature was measured using microcomputer thermometers (JENCO Electronics Ltd., Taipei, Taiwan) right before and immediately after HI. The NF and OF rat pups were the respective control naive pups, while the pups that had experienced HI were defined as the NF-HI and OF-HI groups, respectively.

### Metabolic parameter analysis

P7 NF and OF pups were sacrificed, and the fat pads in the interscapular and perirenal spaces were dissected and weighed. The fat deposit ratio was calculated as follows: (fat pads weight/bodyweight) × 1000. Blood samples were collected before and after HI. The pups were kept in a 30°C incubator for a 1-hour fasting period before blood sampling. Plasma levels of glucose were analyzed using a glucose kit (Biosystem SA, Barcelona, Spain), and insulin was measured using a rat insulin ELISA kit (Mercodia AB, Uppsala, Sweden). Serum levels of free fatty acids (FFAs) were measured using a Wako FFA kit (HR Series NEFA-HR, Wako Chemicals, VA, USA), and triglycerides (TG) were determined with a spectrocolorimetric diagnostic kit (Triglyceride-GPO Reagent Set, Teco Diagnostics, Richmond, VA, USA).

### Brain damage measurement

Brains were removed after perfusion with 4% paraformaldehyde (Sigma-Aldrich, St Louis, MO, USA), embedded in paraffin blocks, and sectioned coronally (10 μm-thick) from the genu of the corpus callosum to the end of the dorsal hippocampus. Brain damage was determined by Nissl staining and TUNEL reaction (TdT-FragEL™ DNA Fragmentation Detection Kit, Calbiochem, Darmstadt, Germany) at 24 hours post-hypoxia (for acute neuronal injury after HI), and also by Nissl staining at P21 (for the protective effect of JNK inhibition) and P85 (for long-term outcome of overweight pups after HI).

One in every twenty sections was stained with cresyl violet. The brain area of bilateral hemispheres was assessed manually by tracing the histological area using a computerized image analysis system (Image-Pro Plus 4.5, Media Cybernetics, Bethesda, MD, USA) linked to a Nikon E400 microscope, and the volume was calculated according to Cavalieri's principle using the formula V = ΣAPt, where V is the total volume, ΣA the sum of the areas measured, P the inverse of the section sampling fraction, and t the section thickness [16]. The percentage of volume loss in the lesioned versus the non-lesioned hemisphere was defined as: (contralateral volume - ipsilateral volume)/contralateral volume.

The histopathology was also determined by TUNEL reaction for neuronal apoptosis 24 hours post-HI. The TUNEL reaction product was visualized with streptavidin-biotin-peroxidase complex and diaminobenzidine at 200X magnification. In each brain, measurement of TUNEL-(+) cells was performed on five visual fields (one visual field = 0.136 mm^2^) in the cortex and three fields in the hippocampus of the five reference planes, which corresponded to plates 15, 18, 27, 31, and 39 in a rat-brain atlas [17]. The numbers of TUNEL-(+) cells were expressed as the average number of TUNEL-(+) cells per visual field.

### Neurobehavioral outcomes measurement by the Morris water maze

The Morris water maze test was performed on P44-P45. A circular pool (160 cm diameter × 50 cm high) divided into four quadrants was filled with water (26 ± 1°C), and an 8 × 8 cm platform was positioned 1 cm below the water surface in the center of one of the quadrants. Four points on the perimeter of the pool were designated and room lights illuminated the pool. On days 1 and 2, rats were given four training sessions (two per day) to escape onto the submerged platform. The quadrant in which the platform was located remained constant, but the point of immersion into the pool varied in a quasi-random order. The time for the rat to escape onto the submerged platform was recorded (escape latency and distance traveled) by a computer program (EthoVision; Wageningen, The Netherlands) connected to a camera mounted in the ceiling directly above the pool, as described previously [14].

### Immunohistochemistry

One or 24 hours after hypoxia, brains were taken after the rats had been perfused with 4% paraformaldehyde (Sigma-Aldrich), and post-fixed overnight at 4°C, followed by incubation with 30% sucrose phosphate buffer for 48 hours. Serial frozen sections (10 μm-thick) were collected on gelatin-coated slides.

BBB permeability measured by IgG extravasation staining was performed 24 hours post-hypoxia. Brain sections were incubated with 0.3% H_2_O_2_/methanol for 30 minutes, and then anti-IgG antibody (HRP-conjugated 1:200; Chemicon, Billerica, MA, USA) for 2 hours. Biotin-peroxidase signals were detected using 0.5 mg/mL 3'3'-diaminobenzidine/0.003% H_2_O_2 _as a substrate. Measurements of the integrated optical density (IOD) of IgG signals in the cortex were analyzed using imaging software (ImagePro Plus 6.0, Media Cybernetics) at 200× magnification per visual field (0.145 mm^2^). The mean IOD was counted and averaged from three visual fields per section, and three brain sections, which corresponded to plates 18, 31 and 39 in a rat-brain atlas [17], of each brain of each experimental group were compared to those of the control group and expressed as relative IOD ratios [18,19].

### Immunofluorescence staining

Immunofluorescence was performed on frozen sections. Activated microglia (ED1) and apoptosis (cleaved caspase-3) were measured at 24 hours post-hypoxia, and pJNK at 1 hour post-hypoxia. Brain sections were blocked with 2% normal goat serum and 0.1% Triton X-100, and probed with primary antibodies p-JNK (1:100, Cell Signaling #9251, #9255, Danvers, MA, USA), cleaved caspase-3 (1:100, Cell Signaling #9661), NeuN (1:200, Chemicon, MAB377), RECA1 (1:100, Abcam, ab9774, Cambridge, MA, USA), GFAP (1:100, Chemicon, MAB360), Iba1 (1:200, Wako 019-19741, Tokyo, Japan), or ED1 (1:100, Chemicon, MAB1435) in PBS/0.03% Triton X-100 at 4°C overnight. The sections were then incubated with Alexa Fluor 488 goat IgG and Alexa Fluor 594 goat IgG (Invitrogen, A-11001, A-11005, A-11008, A-11012, Carlsbad, CA, USA) secondary antibodies for 1 hour at room temperature. Images were acquired on a Nikon E400 fluorescence microscope (Tokyo, Japan). Digitally captured images were analyzed using NIS-Elements imaging software (Nikon, Tokyo, Japan). ED1-(+) microglia were measured at 200× magnification per visual field (0.145 mm^2^) in the cortex, and three visual fields per section, and three brain sections, which corresponded to plates 18, 31 and 39 in a rat-brain atlas [17], of each brain were counted and expressed as an average number per visual field [19].

### Electronic microscopy examination

Twenty-four hours after hypoxia, brains were taken after the rats had been perfused with 2% paraformaldehyde (Sigma-Aldrich) and 2% glutaraldehyde in 0.1 M pH 7.2 phosphate buffer, and postfixed in the same fixative for 2 hours. The samples were blocked and fixed in 1% osmium tetroxide aqueous solution (Sigma-Aldrich) for 1 hour, and washed with ddH2O for 10 min 3 times, then dehydrated in increasingly graded ethanol and pure propylene oxide (Sigma-Aldrich). The samples were embedded in Epon at room temperature and polymerized in an oven at 55°C for 1 day. Eighty nm thick sections were cut and collected onto the grids. The sections were then stained with lead citrate and uranyl acetate and observed with a JOEL 1200 EX transmission electron microscope (JEOL USA, Inc., Peabody, MA).

### Western blot analysis

Ipsilateral cerebral cortices were homogenized in cold lysis buffer, and the protein concentrations were determined using a Bio-Rad Protein Assay kit (Bio-Rad Laboratories, Hercules, CA). Samples (50-100 μg) were separated using 10% SDS-PAGE and blotted onto polyvinylidene fluoride (PVDF) membranes. Membranes were incubated with primary antibodies, and immunoreactivity was detected by horseradish-conjugated secondary antibody and visualized using enhanced chemiluminescence (Amersham, Piscataway, NJ, USA). The following primary antibodies were used: anti-caspase-3 (1:1000; Cell Signaling #9662), anti-poly (ADP-ribose) polymerase (PARP) (1:1000; Cell Signaling #9542), anti-spectrin (1:1000; Chemicon, MAB1622), anti-Grp78 (1:1000; Cell Signaling #3183), anti-phospho-p38 (Thr180/Tyr182) (1:1000; Cell Signaling #9211), anti-JNK (1:1000; Cell Signaling #9258), anti-phospho-JNK (Thr183/Tyr185) (1:1000; Cell Signaling #9251), anti-phospho-c-Jun (Ser63) (1:1000; Cell Signaling #9261), anti-phospho-Bim_EL _(Ser65) (1:1000; Upstate 36-004, Lake Placid, NY, USA), and anti-actin (1:5000; Chemicon, MAB1501). Western blot signals were quantified by scanning with a ScanJet scanner (Hewlett Packard, Palo Alto, CA) and the band intensity was analyzed using Image-Pro Plus software (Media Cybernetics, Silver Spring, MD) [14,15].

### *In Vitro *kinase assay for JNK

JNK activity was measured using a specific kit (Cell Signaling), and glutathione S-transferase (GST)-Jun (1-79) fusion peptides served as the substrate for JNK. In brief, tissue lysates (200 μg) were incubated overnight at 4°C with GST-Jun fusion protein beads. After washing, the beads were resuspended in kinase buffer containing ATP, and the kinase reaction continued for 30 minutes at 30°C. Reactions were stopped by adding polyacrylamide gel electrophoresis sample loading buffer. Proteins were separated by electrophoresis on 10% SDS-PAGE, transferred onto PVDF membranes, and incubated with phospho-c-Jun (Ser63) antibody. Immunoreactivity was detected using enhanced chemiluminescence.

### JNK inhibition

AS601245, a highly specific JNK inhibitor, blocks JNK activity by binding to its ATP-binding site [20]. Rat pups were anesthetized with 2.5% halothane and intracerebroventricularly infused with 100-nmol, 150-nmol or 200-nmol AS601245 (Alexis Biochemicals, Lausen, Switzerland) dissolved in DMSO or vehicle (DMSO, Sigma-Aldrich) into the right cerebral hemisphere 30 minutes prior to HI using a 30 gauge needle with a 10-μl Hamilton syringe (infusion rate 1 μl/min). The pups treated with 200-nmol AS601245 died soon after injection, therefore, 100-nmol and 150-nmol AS601245 were used in this study. The location of the injections in relation to the bregma was 2.0 mm posterior to, 1.5 mm lateral to, and 2.0 mm beneath the skull surface, as described previously [14,15]. Brain damage was measured on P21.

### Statistics

We used a commercial program (SPSS version 13.0; SPSS Institute, Chicago, IL) for the statistical analysis. Continuous data were presented as means ± standard errors of mean (SEM), and analyzed using the Student's *t*-test. Repeated measures in a general linear model and paired t tests were applied to compare escape time during the learning phase of the water maze test. For comparisons of mortalities between groups, we used the chi-square test to estimate odds ratios and 95% confidence intervals. Two-way ANOVA was used to evaluate the protective effect of the JNK inhibitor between groups. *P *< 0.05 was considered statistically significant, and all probabilities were two-tailed.

## Results

### Reducing litter size induced over-weight rat pups

**Figure 1:**
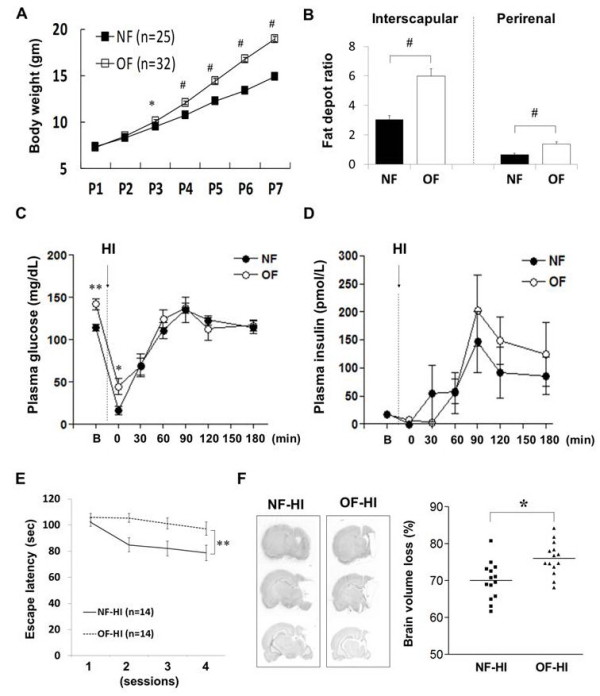
**Metabolic parameters of OF pups and long-term outcomes after HI**. Compared with the control (NF) pups, the overweight (OF) pups had significantly more body-weight from P3 to P7 (**A**), and greater increases of body fat deposit ratios in the interscapular and perirenal areas on P7 (**B**). The OF pups had significantly higher plasma glucose levels than the NF pups before and immediately following HI **(C)**. **(D) **Plasma insulin levels showed no difference between the two groups before and after HI. n = 5-8 in each group at each time point. (**E**) Morris water maze test performed on P44-P45 showed that the NF-HI rats made progress with significantly less escape latency finding the submerged platform during learning than the OF-HI rats. (**F**) The OF-HI rats had significantly more brain volume loss than the NF-HI rats (right panel). B: basal state. *p < 0.05, **p < 0.01, #*p *< 0.001, Data are means ± SEM.

The OF pups were heavier in body-weight than the NF pups from P3 to P7 (Figure 1A). On P7, the OF pups had significant increases of body fat mass (relative to body weight) in the interscapular (5.3 ± 0.4 vs. 3.6 ± 0.3, *p *< 0.001) and perirenal areas (1.13 ± 0.08 vs. 0.74 ± 0.04, *p *< 0.001) compared to the NF pups (Figure 1B). The OF pups also had significantly (*p <*0.01) higher plasma levels of glucose than the NF pups. The levels of plasma insulin, serum free fatty acid and triglycerides were similar between the OF and NF groups (Table 1).

**Table 1:** Metabolic parameters in the plasma of the control and overweight rat pups on postnatal day 7

	Control (NF) rats (n = 10)	Overweight (OF) rats (n = 9)
Glucose (mg/dL) **	114.7 ± 4.1	142.3 ± 6.5
Insulin (pmol/L)	17.9 ± 1.9	17.2 ± 1.1
Free fatty acids (mmol/L)	0.73 ± 0.03	0.75 ± 0.07
Triglycerides (mmol/L)	1.27 ± 0.14	1.45 ± 0.18

** *P *value < 0.01, Data are means ± SEM

### Rat pups from a small-litter size had more hypoxic-ischemic brain injuries and worse neurobehavior performances at adulthood

The mortality rate during HI was significantly higher in the OF-HI pups (41%, 13/32) than in the NF-HI pups (8%, 2/25) (odds ratio 7.86, 95% confidence interval 1.58 to 39.3, *p <*0.01). Both groups had comparable body temperatures before HI (OF: 34.8 ± 0.3°C vs. NF: 34.5 ± 0.5°C) and immediately after HI (OF-HI: 33.6 ± 0.8 °C vs. NF-HI: 33.6 ± 1.0°C). The plasma levels of glucose decreased substantially immediately following HI, and returned to basal levels one hour post-HI in both NF-HI and OF-HI pups (Figure 1C). The OF-HI pups had significantly higher plasma levels of glucose only at the time point immediately post-HI than the NF-HI pups (*p <*0.05). Both groups had similar plasma levels of insulin before and after HI (Figure 1D). The Morris water maze task was carried out on P44-P45, and it showed that the NF-HI rats made progress and gradually reduced escape latency from session 1 to session 4 (102.2 ± 3.2 seconds in trial 1 vs. 78.6 ± 6.1 seconds in trial 4, *p *< 0.001) during learning, but the OF-HI rats did not make progress (105.8 ± 3.1 seconds in trial 1 vs. 97.2 ± 5.1 seconds in trial 4, *p *> 0.05) (Figure 1E). The total escape latency between the two groups was significantly different (*p *< 0.01, F = 7.301). The long-term pathological outcome on P85 showed that the OF-HI rats had significantly more brain volume loss than the NF-HI rats (*p <*0.05) (Figure 1F).

### Rat pups from a small-litter size had aggravated apoptosis, microglia activation and blood brain barrier damage after hypoxic ischemia

**Figure 2:**
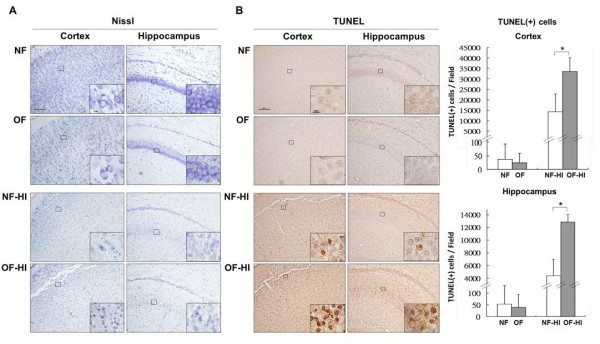
**Neuropathological examination**. **(A) **Nissl staining showed no difference between the NF pups and OF pups in the cortex and hippocampus on P7, but 24 hours post-HI, the OF-HI pups had more neuronal damage than the NF-HI pups. **(B) **TUNEL staining revealed that the OF-HI pups had significantly more TUNEL-(+) cells in the cortex and hippocampus than the NF-HI pups. n = 4. *p <0.05. Scale bar = 100 μm (A, B), and = 10 μm in the insets (A, B).

**Figure 3:**
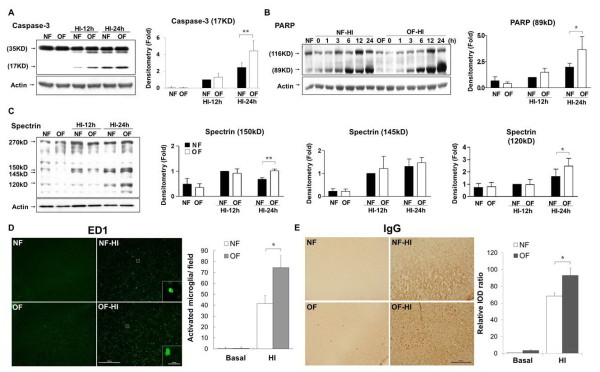
**Apoptosis, activated microglia and BBB damage after HI**. Twenty-four hours post-HI, western blots showed that the OF-HI pups had significantly increased levels of cleaved caspase-3 **(A) **and PARP **(B) **in the cortex compared with the NF-HI pups. The OF-HI pups also had significant increases of 150kD and 120kD a-spectrin fragments than the NF-HI pups **(C)**. n = 4-5 in each group at each time point. Immunohistochemistry showed that the OF-HI pups had significant increases of ED1-(+) activated microglia **(D)**, and more extravasation of IgG **(E) **indicating BBB leakage 24 hours post-HI compared to the NF-HI pups. The representative pictures in **D **and **E **were from the ipsilateral cortex of plate 31 of the rat-brain atlas [17]. Scale bar = 100 μm in low magnifications (**D, E**), and 20 μm in high magnifications (**D**). *p < 0.05, **p < 0.01, Data are means ± SEM.

**Figure 4:**
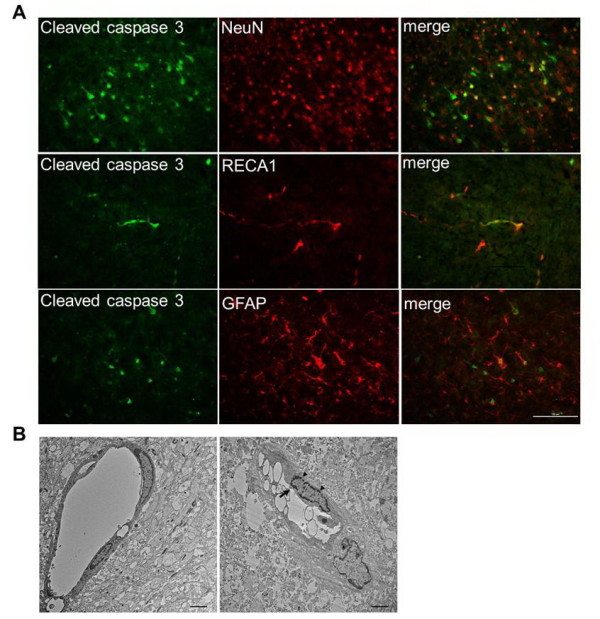
**Apoptosis of neurons and vascular endothelial cells**. (**A**) Immunofluorescence study of the cortex of the OF pups 24 hours post-HI showed that cleaved caspase 3 was expressed mainly in neurons (NeuN) and vascular endothelial cells (RECA1), but very little in astrocytes (GFAP). n = 3 experiments. Scale bar = 100 μm. (**B**) Electronic microscope examination of vascular endothelial cells revealed normal endothelial cells in the OF pups (left panel), and early apoptotic changes, which included condensed and fragmented nuclei (arrowheads) and bleb-like cytoplasm (arrow) in the endothelial cells 24 hours post-HI in the OF-HI rats (right panel). Scale bar = 2 μm. The representative pictures were from the ipsilateral cortex in plate 31 of the rat-brain atlas [17].

Nissl and TUNEL staining showed that the OF pups had similar histological findings as the NF pups on P7 (Figure 2A, 2B). On P8, 24 hours post-HI, the OF-HI pups showed increased neuronal loss (Figure 2A) and had more TUNEL-(+) cells (Figure 2B) in the cortex and hippocampus than the NF-HI pups. Western blots revealed that the OF-HI pups had significant increases of cleaved caspase-3 (*p <*0.01) and PARP (89kD) (*p *< 0.05) levels in the cortex compared to the NF-HI pups 24 hours post-HI (Figure 3A and 3B). Spectrin, a membrane cytoskeleton protein in neurons, undergoes proteolysis mediated by calpain and caspase-3 following HI [21]. 120kD and 150kD α-spectrin fragments are products of caspase-3 cleavage, while the 145kD fragment is due to calpain cleavage [22]. Compared to the NF-HI pups, the OF-HI pups showed significant increases of 150kD (*p *< 0.01) and 120kD (*p *< 0.05) but not 145kD α-spectrin fragments 24 hours post-HI (Figure 3C). Resting microglia were identified as "ramified" microglia with long processes, while primed/activated microglia were identified as microglial cells that were more rounded, with retracted and shorter processes [23]. Immunohistochemistry showed that the OF-HI pups had significantly more ED1-(+) activated microglia (Figure 3D) and increased extravasation of IgG (Figure 3E) in the cortex 24 hours post-HI compared to the NF-HI pups. Immunofluorescence of the cortex of the OF-HI rats showed that activated-caspase 3 expression was found mainly in NeuN-(+) neurons and RECA1-(+) vascular endothelial cells, but very few in GFAP-(+) astrocytes 24 hours post-HI (Figure 4A). Further electronic microscope examination of vascular endothelial cells revealed a normal configuration in the OF pups (Figure 4B, left panel), and early apoptotic changes, which included condensed and fragmented nuclei and bleb-like cytoplasm, of the endothelial cell 24 hours post-HI in the OF-HI pups (Figure 4B, right panel). These findings suggest that small litter size-induced overweight aggravated HI brain damage was associated with upregulation of HI-induced neuronal apoptosis, microglial activation, and endothelial and BBB damage in the rat pups.

### Rat pups from a small-litter size had JNK hyperactivation in neurons, microglia and endothelial cells after hypoxic-ischemia

**Figure 5:**
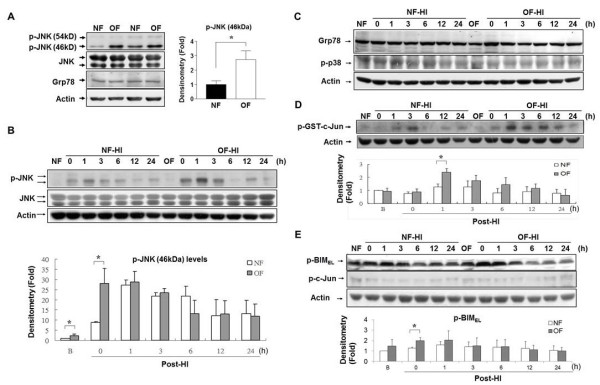
**JNK hyperactivation in the NF and OF pups**. **(A) **Western blots showed significantly higher p-JNK but not Grp78 levels in the OF pups compared to the NF pups on P7. After HI, the OF-HI pups also had significantly higher p-JNK **(B) **but not Grp78 or phospho-p38 (p-p38) (**C**) levels immediately post-HI than the NF-HI pups. **(D) ***In vitro *kinase assay of JNK showed significant increases of p-GST-c-Jun levels one hour post-HI in the OF-HI pups. **(E) **The OF pups had higher levels of p-Bim_EL _instead of p-c-Jun immediately post-HI than the NF pups. n = 4~5 in each time point of each group, *p < 0.05, Data are means ± SEM.

Endoplasmic reticulum stress and hyperactivation of JNK plays important roles in obesity and ischemic brain injury in adult rats [10]. On P7, the OF pups had significant increases of phospho-JNK (p-JNK), but not endoplasmic-reticulum chaperon protein Grp78 levels compared to the NF pups (*p <*0.05, Figure 5A). HI induced rapid and sustained increases of p-JNK levels in both OF-HI and NF-HI groups. The OF-HI pups exhibited higher p-JNK levels (*p *< 0.05) immediately post-HI than the NF-HI pups (Figure 5B). There were no differences in the Grp78 or phospho-p38 (p-p38) levels post-HI between the OF-HI and NF-HI groups (Figure 5C). *In vitro *kinase assays confirmed that the OF-HI pups had higher phosphorylated GST-cJun (p-GST-cJun) levels than the NF-HI pups one hour post-HI, confirming early upregulation of JNK activity after HI in the OF group (Figure 5D). Next, we examined two potential downstream molecules of JNK, Bim_EL _and c-Jun. The OF-HI pups had higher levels of Ser65 phosphorylation of Bim_EL _soon after HI than the NF-HI pups, while the phospho-c-Jun levels did not differ between the two groups (Figure 5E). These findings suggest that JNK hyperactivation after HI might worsen brain damage in overweight pups.

**Figure 6:**
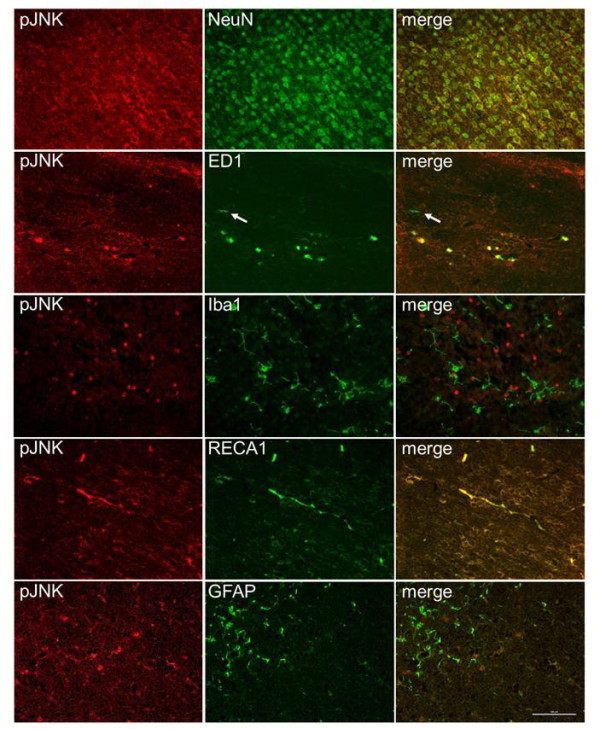
**pJNK expression in neuron, microglia and vascular endothelial cell**. Immunofluorescence of p-JNK one hour post-HI in the OF-HI pups showed that p-JNK was mainly expressed in neurons (NeuN), most of the round-shaped activated microglia (ED1) and vascular endothelial cells (RECA1), but very little in resting microglia (Iba1) or astrocytes (GFAP). Arrows indicate resting ramified ED1+ microglia that did not express pJNK. The representative pictures were taken from the ipsilateral cortex in plate 31 of the rat-brain atlas [17]. n = 3 experiments. Scale bar = 100 μm.

Further immunofluorescence staining in the OF-HI group one hour after HI confirmed that p-JNK was expressed mainly in the neurons that co-expressed NeuN, and in the vascular endothelial cells that co-expressed RECA1, but not in the astrocytes that showed GFAP (Figure 6). About 76 ± 19% of the round-shaped ED1-(+) activated microglia expressed p-JNK. In contrast, only 5 ± 3% of resting microglial cells (ramified Iba1+ microglia with long processes) expressed p-JNK [23]. These findings suggest that neonatal overweight may aggravate HI brain damage through JNK hyperactivation in neurons, microglia and vascular endothelial cells.

### JNK inhibition reduced apoptosis, microglial activation, BBB leakage and brain damage after hypoxic ischemia in rat pups from a small-litter size

**Figure 7:**
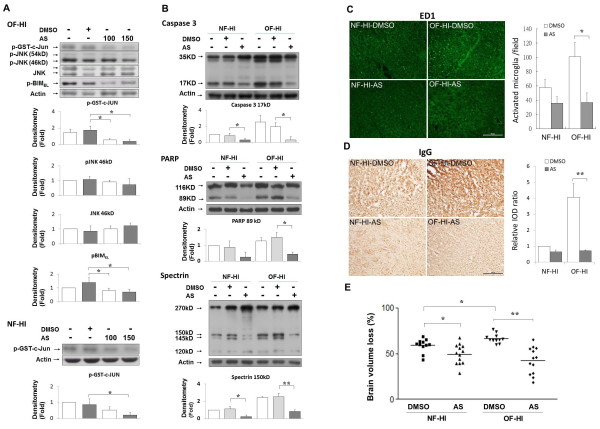
**JNK inhibition against HI in the NF and OF pups**. **(A) **Intracerebroventricular injections of AS601245 (AS, 100-nmol, 150-nmol) effectively diminished JNK activity one hour post-HI in the OF-HI and NF-HI pups. AS injections decreased p-Bim_EL _levels but did not affect p-JNK levels in the OF-HI pups. n = 4 experiments. **(B) **Compared with their respective vehicle-treated pups, JNK inhibition (150-nmol AS) caused more reduction of cleaved levels of caspase-3 and PARP, and spectrin fragments in the OF-HI pups than in the NF-HI pups. AS 150-nmol treatment significantly decreased ED1-(+) microglia **(C) **and IgG extravasation **(D) **in the cortex 24 hours post-HI in the OF-HI but not in the NF-HI pups. n = 4-5 experiments. The representative pictures were from the ipsilateral cortex in plate 31 of the rat-brain atlas [17]. **(E) **Brain-volume loss measured on P21 showed the more protective effects of AS in the OF-HI compared to the NF-HI pups. Scale bar = 100 μm. *p < 0.05, **p < 0.01. Data are means ± SEM.

To determine the worsening effect of JNK hyperactivation on HI brain injury in the OF pups, we inhibited JNK activation with a specific ATP competitor (AS601245) in the NF and OF pups before HI [20,24]. Compared with DMSO, 100-nmol and 150-nmol AS601245 effectively diminished JNK activity in both NF-HI and OF-HI pups (Figure 7A). AS601245 injection significantly reduced the p-Bim_EL _levels but not the p-JNK levels in the OF-HI group, further implicating the interaction between JNK and Bim_EL_. Compared with the respective vehicle-treated pups, JNK inhibition (150-nmol AS601245) caused more attenuation of the cleaved levels of caspase-3 and PARP, and the α-spectrin fragments in OF-HI pups compared to the NF-HI pups (Figure 7B). Immunohistochemistry showed that JNK inhibition also caused a significant reduction of HI-induced ED1-(+) activated microglia (Figure 7C) and IgG extravasation (Figure 7D) in the OF-HI pups but not in the NF-HI pups. AS601245 significantly reduced the brain volume loss in NF-HI, and especially in OF-HI pups (both *p *< 0.05 versus DMSO-treated rats) (Figure 7E). There was a significant interaction between OF and AS601245 effects [two-way ANOVA, *F*(1,44) = 5.858, *p *= 0.02], indicating JNK inhibition was more protective in OF-HI than in NF-HI pups.

## Discussion

In this study, we showed that rat pups from a small litter size from P1 to P7 had increased susceptibility to HI injury on P7, evidenced by increased HI mortality, and worsened neurobehavioral performance and aggravated brain injury in long-term follow up. The aggravated HI brain injury in the OF rat pups was associated with JNK hyperactivation in neurons, microglia and vascular endothelial cells one hour post-HI, and also with upregulation of neuronal apoptosis, microglial activation and BBB leakage 24 hours post-HI. JNK inhibition reduced apoptosis, microglial activation and BBB damage after HI, and decreased HI brain injury, especially in the OF pups. These findings suggest that the overweight rat pups from a small litter size had increased HI-induced neuronal apoptosis, microglial activation and BBB damage, and aggravated brain damage through JNK hyperactivation.

Two methods, maternal nutritional excess and overfeeding during the suckling period, are commonly used to study the effect of metabolic programming on rodent pups. Maternal nutritional excess, such as high fat or cholesterol intake during pregnancy and the lactation period, results in a rat offspring phenotype that closely resembles human metabolic syndrome in adulthood [25,26]. The effects of maternal nutritional excess on body-weight or adiposity in the perinatal period of the offspring vary with the type and timing of the diet program. Overfeeding by litter size reduction increases milk availability during the suckling period and subsequently induces overweight pups [12,13,27]. We defined the NF pups as 12 pups per dam because Sprague-Dawley rats are commonly maintained in a litter of ten to 12 during the pre-weaning period [27]. We exploited the effect of litter size culling to induce early-onset overweight in P7 pups, and defined OF rats by reducing the litter size to 6 pups per dam, and NF rats as 12 pups per dam starting from P1. Indeed, the OF pups gained significantly more body-weight and fat mass depots on P7 as compared to the NF pups.

The effect of litter size on HI brain damage has been reported in two previous studies [28,29]. In Trescher's study [28], newborn rats were raised in a litter of 6 (well-nourished) or 14 (under-nourished) pups from P2. They found that the well-nourished rat pups had more HI brain damage than the under-nourished pups. In Oakden's study [29], rat pups culled to 10 pups per dam on P2 were heavier and showed more severe brain damage than pups from birth-sized litters (13-15 pups). Both studies found that heavier animals were more susceptible to HI, but the importance of being overweight from a small-litter size was not taken notice of. We demonstrated that JNK hyperactivation in neurons, microglia and vascular endothelial cells plays an important role in overweight-aggravated HI injury in the neonatal brain.

Apoptosis accounts for higher HI susceptibility of the developing brain [2,3]. We found that the OF pups had more TUNEL-(+) cells, and increased caspase-3 and PARP cleavage levels post-HI than the NF pups. These findings suggest that increased apoptosis is associated with the aggravation of HI neuronal damage in overweight rat pups. One of the events to occur after HI in the neonatal brain is the appearance of abundant numbers of activated microglia, which peaks at 1-4 days post-HI [21,30]. Activation of microglia through Toll-like receptor 4 exacerbates neuronal damage [8,30], and inhibiting microglial activation reduces HI injury [31]. Vascular endothelial cell injury and BBB damage also play important roles in neonatal brain injuries [9,15,18]. Extensive BBB disruption with maximum IgG immunoreactivity occurs at 24 hours, followed by significant brain injury at 7 days post-insult [18]. The vulnerability of vascular endothelial cells and BBB may be related to the activation of microglia, which contributes to BBB disruption through matrix protease generation [32,33]. Recruiting activated leukocytes to the injured cerebrum through damaged BBB may result in sustained activation of microglia, which, in turn, may produce further cerebral damage through prolonged production of inflammatory cytokines [34,35]. Compared with the NF group, the OF group had more microglial activation and BBB damage in the cortex post-HI. These findings suggest that increases of BBB permeability may act in concert with microglia activation to further accentuate brain injury. Taken together, overweight in pups aggravates HI brain injury in association with more neuronal apoptosis, microglia activation and BBB leakage, the three critical mechanisms involved in the evolution of neonatal HI brain injury.

Extravascular IgG immunoreactivity in the cortex after HI can be observed at cellular as well as parenchymal levels. IgG entry into neurons after brain ischemia has been described in studies using immunostaining [36-38]. This effect is presumably related to membrane damage in injured neurons which permits the influx of various proteins, or increased incorporation of extravasated serum proteins in surviving neurons [36]. Glia can also rapidly take up plasma proteins from the extracellular space of the injured brain through endocytosis [38]. Fc-receptors on reactive microglia can trap IgG in the tissue and thus facilitate its phagocytic activity [39]. In addition, extravasated plasma constituents after transient cerebral ischemia might act also as an inductive factor on microglial cells [40].

JNK is known to be activated in response to stress and ischemia, and has recently emerged as a central regulator in the development of insulin resistance in obesity [10,11]. It is established that feeding mice a high-fat diet causes activation of JNK. Moreover, *JNK *knockout mice are protected against the effects of high-fat diet-induced insulin resistance [10]. These observations indicate that JNK plays a critical role in the metabolic stress response of obesity. Tumor necrosis factor-alpha, free fatty acid and reactive oxygen species are potent JNK activators [10,41]. Our finding that the OF pups had significantly higher levels of p-JNK levels before and after HI compared to the NF pups suggests that an excess volume of fat in the OF pups may contribute to JNK hyperactivation. Since the blood levels of free fatty acid was not elevated in the OF pups, further studies are needed to address whether inflammatory cytokines and oxidative stress occur and account for JNK hyperactivation in OF pups from a small-litter size.

Activation of JNK signaling pathways leads to c-Jun-mediated inflammatory cytokine production [42,43], and proapoptotic death signaling events [44,45]. In vitro studies have shown that JNK/p38-MAPK signaling is the predominant pathway for cytokine production from LPS-stimulated or hypoxia-exposed microglia [46,47]. JNK signaling has also been shown to be involved in subarachnoid hemorrhage-associated BBB disruption and stress-induced apoptosis of cerebral vascular endothelial cells [48,49]. Therefore, JNK signaling may be a shared pathway involved in the stress responses of neurons, microglia and vascular endothelial cells. Our finding that JNK was activated in the cortex of P7 OF pups suggests that being overweight in the neonatal period induces a metabolic stress response in the brain. In addition, JNK was hyperactivated in the neurons, microglia and vascular endothelial cells post-HI in the OF pups, and inhibition of JNK activation reduced HI-induced neuronal apoptosis, decreased microglia activation and attenuated BBB damage in the OF pups. These findings suggest that OF may induce a programming effect on the neurons, microglia and vascular endothelial cells of the neonatal brain through JNK hyperactivation after HI.

JNK exerts a pro-apoptotic function in stroke models of adult animals by direct phosphorylation of the downstream molecules, c-Jun and Bim_EL_. Our finding that the increased p-JNK levels after HI correlated with the increased phosphorylated Bim_EL _levels indicates that JNK hyperactivation in the overweight pups may exacerbate pro-apoptosis pathways and aggravate brain damage through Bim_EL _signaling. Inhibition of JNK activity has been shown to be neuroprotective in adult models of global ischemia and focal ischemia [20,42], and JNK inhibition in middle-cerebral-artery occlusion stroke models has been shown to attenuate apoptosis and decrease brain infarct size [45,50]. We found that intracerebroventricular injections of JNK inhibitor AS601245 not only inhibited JNK activity and reduced Bim_EL _phosphorylation after HI, but also significantly reduced HI brain injury in the NF-HI and OF-HI rat pups. More importantly, the neuroprotective effect of JNK inhibition was significantly greater in the OF-HI pups. These findings provide further evidence that hyperactivation of JNK-Bim_EL _signaling after HI may be involved in overweight-aggravated brain damage of neonatal rats.

Ginet et al. [51] recently showed that D-JNKI1, which interferes with JNK signaling through inhibiting the transcription of c-fos, did not reduce HI brain volume loss in neonatal rats. We found that HI induced a rapid increase of p-JNK and JNK activities immediately after HI, and that inhibition of JNK activities by AS601245 significantly reduced brain volume loss in both NF-HI and OF-HI rats. The reason for the discrepancy remains unknown, but it may be related with the difference in the type of JNK inhibitors used, and the route and schedule of JNK inhibitors that were administered. We used a single intracerebroventricular injection of AS601245 30 minutes prior to HI, while Ginet et al. administered repeated intraperitoneal injections of D-JNKI1 30 minutes before HI, and 3, 5, 8, 12, and 20 hours after HI. Instead of using D-JNKI1 (a small peptide inhibitor), we chose a specific JNK inhibitor AS601245 (a small molecule inhibitor) which directly decreases JNK activities. Our results are consistent with a recent study showing that neonatal mice lacking *JNK3 *were protected against cerebral HI [52].

Obesity is associated with chronic inflammatory responses characterized by abnormal production of cytokines and oxidative stress [53-55]. Fat tissue is a key endocrine organ and has a central role in obesity-associated complications. Macrophages tend to accumulate in adipocytes in direct proportion to the size of adipocyte [55]. In turn, infiltrating inflammatory macrophages can produce reactive oxygen species and inflammatory cytokines, such as tumor necrosis factor-alpha [56]. Obesity has been related to oxidative stress [53]. It is known that the cytokines and reactive oxygen species released from fat tissue have the ability to affect other tissues such as the liver, heart and brain [57]. Furthermore, hypoxia is associated with an increased expression of inflammatory genes in adipose tissue of obese mice [58]. A recent study on mice and human adipocytes reported that hypoxia led to the stimulation of the expression and secretion of cytokines [59]. That is, hypoxia may stimulate inflammatory responses via macrophages. The brain is an immunologically active organ, and has indirect communication with the immune and endocrine systems. Thus, systemic inflammatory reactions and oxidative stress responses can influence brain function [60]. Therefore, it is possible that increases of fat tissue may contribute to more neuronal loss, microglial activation, and endothelial cell and BBB damage in OF pups after HI via upregulation of oxidative stress and inflammation.

Neuronal apoptosis and death occur progressively after HI in rat pups [2,3]. The higher mortality during HI in the OF compared to the NF pups suggests that poorer cardiovascular or pulmonary responses rather than increases of brain injuries occurs in OF pups during hypoxic insult. The mechanism of poor cardiovascular and respiratory function in OF pups during hypoxia remains to be examined. Hyperglycemia has been shown to worsen ischemic outcome in various adult animal models of global and focal cerebral ischemia [61,62]. In contrast, Vannucci showed that pretreatment with glucose before HI reduced the severity of brain damage in neonatal rats [63]. Whether the slight increase of blood glucose level attributed the increased brain injury in OF-HI pups remains to be elucidated. Further studies are also needed to examine whether high glucose levels and an increased fat volume have a synergistic effect on the development of increased infarct volume after HI in OF pups.

**Figure 8:**
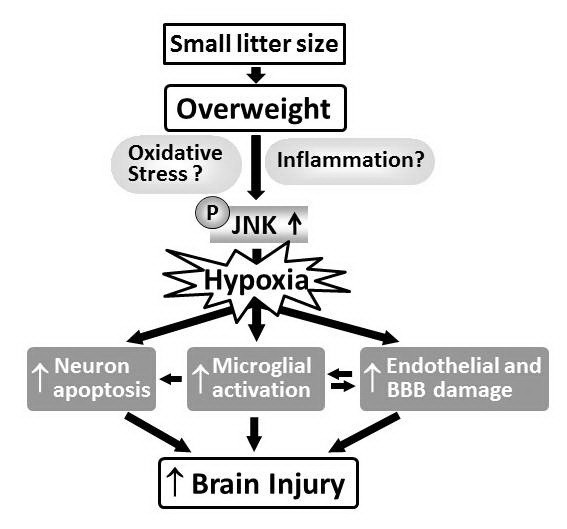
**The proposed diagram**. A diagram showing that JNK hyperactivation in the neurovascular unit (neurons, endothelial cells and microglia) after HI may be the potential link between being overweight from a small litter size and worsened HI injuries in the neonatal brain.

The neurovascular unit, composed of neurons, microvessels and microglia, is considered a major target of ischemic-reperfusion injury [64,65]. Dysfunction of the neurovascular unit may further disrupt microcirculation and hence promote progression of the ischemic lesion. The findings that the OF-HI group had more HI-induced neuronal apoptosis, vascular endothelial cells and BBB damage, and microglial activation compared to the NF-HI group suggest that the neurovascular unit is more susceptible to HI injury in OF pups. A proposed diagram (Figure 8) is provided to show that JNK hyperactivation in the neurovascular unit (neurons, endothelial cells and microglia) after HI may be the potential link between being overweight from a small litter size and worsened HI injury in the neonatal brain. Our findings are consistent with a clinical report that evaluated the factors determining the treatment efficacy of head cooling hypothermia in newborns with HI encephalopathy [66]. The study found that larger infants (birth weights of ≥ 25^th ^percentile) displayed a lower frequency of favorable outcomes in the control group, but a greater improvement with cooling. The adverse effect of a greater birth-weight in the control infants remained significant even after adjustment for the severity of encephalopathy. The clinical and animal findings unequivocally demonstrate that large-for-gestational-age newborns or OF pups have worse neurological outcome following HI than appropriate-for-gestational-age newborns or NF pups.

## Conclusions

We found that rat pups from a small-litter size showed increased vulnerability to hypoxia. This effect may be related to increased body weight. JNK activation may be a shared signaling pathway that underlies overweight-induced stress responses in neurons, microglia and vascular endothelial cells in the neonatal brain. Neonatal overweight induced by reduced litter size aggravated HI brain injuries in the rat pups through JNK hyperactivation. JNK hyperactivation may be an important step in signal transduction underlying why being overweight exacerbates HI injury in the neonatal brain.

## Abbreviations

BBB: Blood-brain barrier; HI: Hypoxic-ischemia; JNK: c-Jun N-terminal kinase; OF: Overweight; NF: Control; PARP: Poly (ADP-ribose) polymerase; IOD: Integrated optical density.

## Competing interests

The authors declare that they have no competing interests.

## Authors' contributions

YFT and HCW participated in the design of the study and performed the statistical analysis. YST provided continuous intellectual input, and YFT evaluated and interpreted of data. LWW and CJH provided technique support for animal preparation and carried out the immunoassays. CCH conceived, designed and coordinated the project, and drafted the manuscript. All authors read and approved the final manuscript.
